# “Locus minoris resistentiae” and connective tissue weakness in older women: a case report and literature review on pelvic organ prolapse with inguinal bladder hernia

**DOI:** 10.1186/s12905-021-01554-4

**Published:** 2021-12-20

**Authors:** A. Esber, A. Kopera, M. P. Radosa, I. B. Runnebaum, H. K. Mothes, A. R. Mothes

**Affiliations:** 1 Department of Gynecology, St. Georg Hospital Eisenach, Academic Teaching Hospital, University of Jena, Jena, Germany; 2 Department of Gynecology, Helios Meiningen, Academic Teaching Hospital, University of Jena, Jena, Germany; 3 Department of Gynaecology and Obstetrics, Hospital Bremen North, Bremen, Germany; 4 Women’s University Hospital of Jena, Jena, Germany; 5 Department of General, Visceral and Vascular Surgery, Jena University Hospital, Jena, Germany; 6 Department of Surgery, Sophien-Hufeland-Hospital Weimar, Academic Teaching Hospital, University of Jena, Jena, Germany; 7 Department of Gynaecology, University-Teaching Hospital Eisenach of University Women’s Hospital Jena (Director: Prof. I.B. Runnebaum), Mühlhäuser Str. 94, 99817 Eisenach, Germany

**Keywords:** Inguinal bladder hernia, Pelvic organ prolapse, Connective tissue weakness, Locus minoris resistentiae, Case report

## Abstract

**Background:**

Conditions such as genital prolapse and hernia are known to be related to connective tissue dysfunction. In this report on cases of the rare simultaneous finding of large genital prolapse and post-prolapse repair female inguinal bladder hernia, we aim to contribute to the discussion of a possible clinical definition of connective tissue weakness, for its clinical assessment and preoperative patient counselling.

**Case presentation:**

Three cases of medial third-grade (MIII, Aachen classification) inguinal bladder hernia developing or enlarging after successful stage-IV pelvic organ prolapse (POP) repair at a university pelvic floor centre are presented. All patients were aged ≥ 80 years with long-standing postmenopausal status. One patient was followed for 5 years and two patients were followed for 6 months. In all patients, ultrasound revealed that the hernia sac contained the urinary bladder, which had herniated through the inguinal hernia orifice. A literature search revealed only one case report of direct female inguinal bladder hernia and few investigations of the simultaneous occurrence of POP and hernia in general.

**Conclusion:**

The simultaneous occurrence of inguinal hernia and female POP can lead to bladder herniation following prolapse surgery in the sense of a “locus minoris resistentiae”. Clinical examination for simultaneous signs of connective tissue weakness and counselling prior to pelvic reconstructive surgery could help to increase patients’ compliance with further surgical treatment for hernia.

## Background

As life expectancy in western nations is increasing, issues related to quality of life and physical and mental well-being are of increasing importance for elderly adults and for society [1]. In this context, the investigation of conditions related to tissue ageing and weakness, such as hernia and female genital prolapse, is considered to be of relevant scientific interest.

Inguinal hernias containing the urinary bladder are rare, accounting for 1–5% of all inguinal hernias [2]. They usually occur in obese men aged > 50 years [3], and are encountered infrequently in women [4].

Connective tissue weakness in combination with advanced age and menopausal hormone deficiency are well-known risk factors for pelvic organ prolapse (POP) [5–8]. However, objective clinical criteria for the characterisation of connective tissue weakness are lacking. The simultaneous identification of multiple conditions associated with connective tissue weakness in the same patient could be used as a clinical marker [9–11]. Studies of POP risk constellations have considered the simultaneous occurrence of POP and varicose veins, haemorrhoids and/or hernia, and scoring systems have been proposed [5, 6, 11]. Growing evidence indicates that similar molecular collagen alterations occur in patients with POP and those with hernias [12, 13]. Both conditions are based anatomically on a “locus minoris resistentiae” (Latin for “place of lesser resistance”) [14].

Herein, we present three cases of stage IV POP treated successfully with reconstructive surgery, with the subsequent development or enlargement of medial third-grade (MIII, Aachen classification [15]) inguinal hernias containing the urinary bladder. Our report and literature review are intended to contribute to a possible clinical definition of connective tissue weakness based on the finding of multiple conditions considered to be related to connective tissue alterations, such as female genital prolapse and hernia. The early identification of connective tissue weakness enables the immediate initiation of conservative pelvic-floor treatment. In presenting these cases, we aim to raise awareness of the rare condition of female bladder hernia developing after reconstructive pelvic-floor surgery, to aid its clinical assessment and preoperative patient counselling.

## Case presentation

Data and photographs for the three cases presented were collected from the patient files of the University Pelvic Floor Centre, University Women’s Hospital, Jena, Germany. The data were recorded between 2010 and 2015. All patients provided informed consent prior to examination, surgery and documentation. All patients were women aged ≥ 80 years, with long-standing postmenopausal status and thus hormone deficiency. One patient was followed for 5 years, and the other two patients were followed for 6 months.

### Case 1

**Fig. 1:**
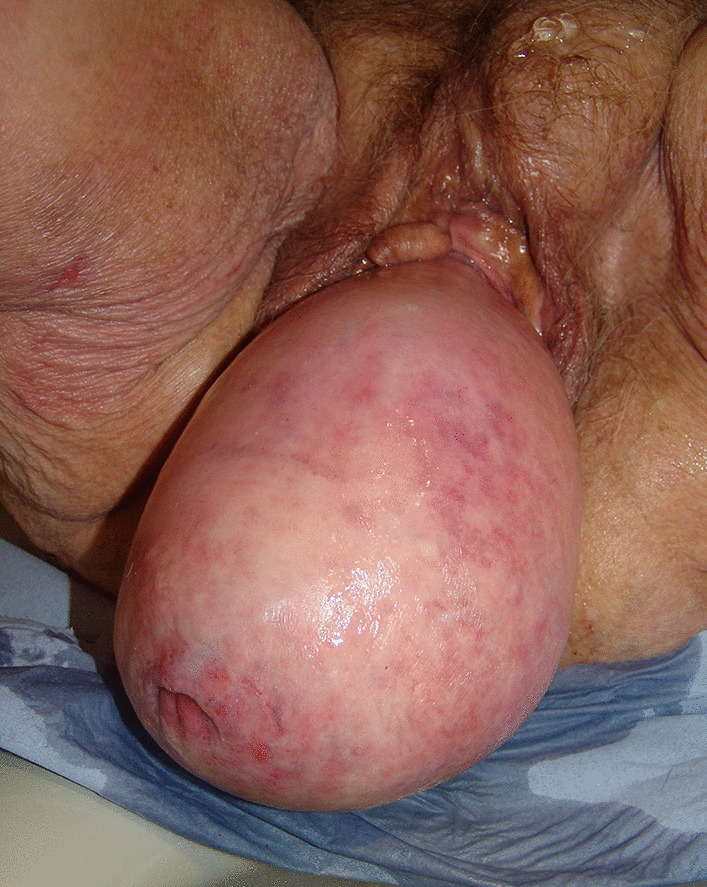
Stage IV genital prolapse and pre-existing inguinal hernia

**Fig. 2:**
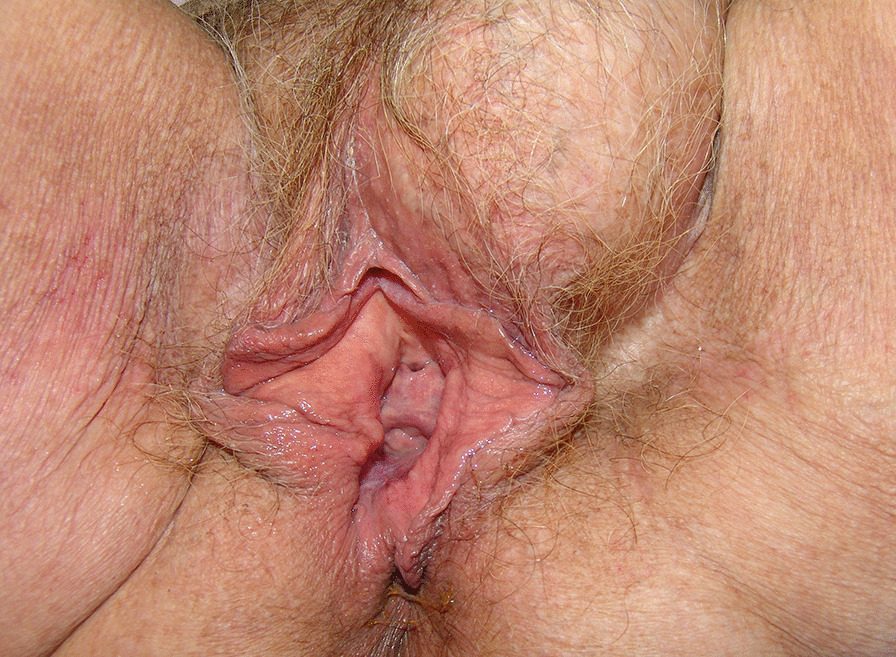
Enlarged inguinal hernia at follow up 3 months after reconstructive surgery for POP

**Fig.3:**
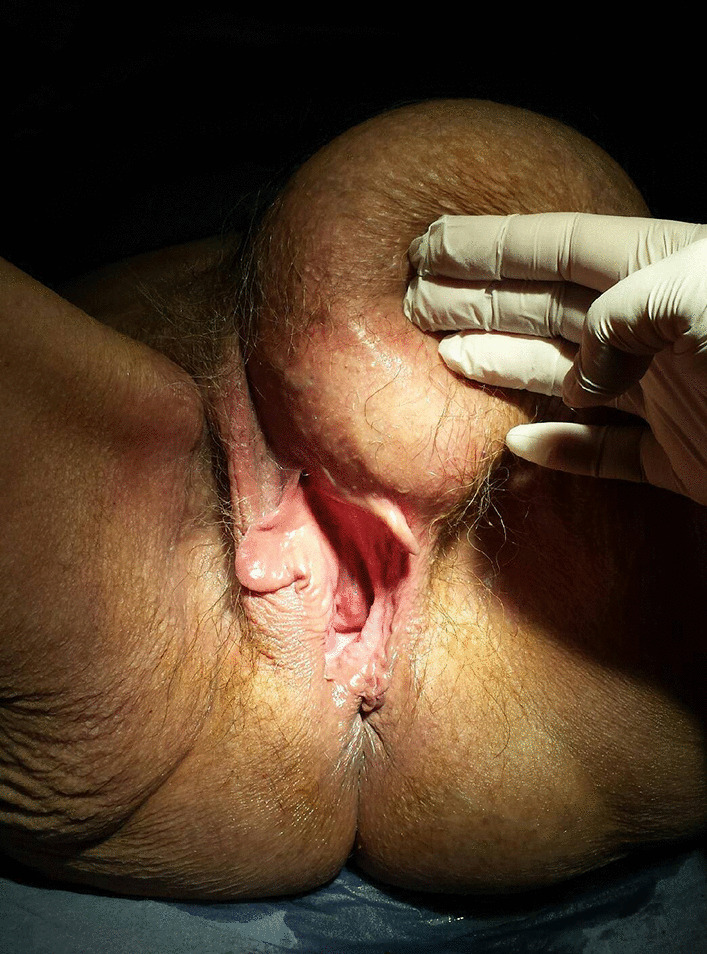
Inguinal bladder hernia at follow up 5 years after reconstructive surgery for POP

An 80-year-old woman presented with an extraordinarily large stage IV genital prolapse and a small, asymptomatic pre-existing inguinal hernia in August 2010 (Fig. 1). She underwent laparoscopic hysterosacropexy in October 2010. Due to the size of the prolapse and the intent to keep the operating time as short as possible, a direct suturing technique was used at the promontory site without mesh implant placement. Secondary anterior repair was performed in November 2010. The inguinal hernia was found to be enlarged on postoperative follow-up examinations conducted at the immediate postoperative period and at 3 months (Fig. 2). Despite her advanced age, the patient attended a 5-year follow-up visit in 2015, where she presented with progressive enlargement of the inguinal hernia, classified as MIII in the Aachen system [15]. On ultrasound, the hernia sac was found to contain the urinary bladder. The urinary bladder had herniated through the inguinal hernia orifice following the POP surgery (Fig. 3). The patient refused surgical treatment of the hernia, which was treated with a binder, yielding a subjectively good outcome.

### Case 2

**Fig. 4:**
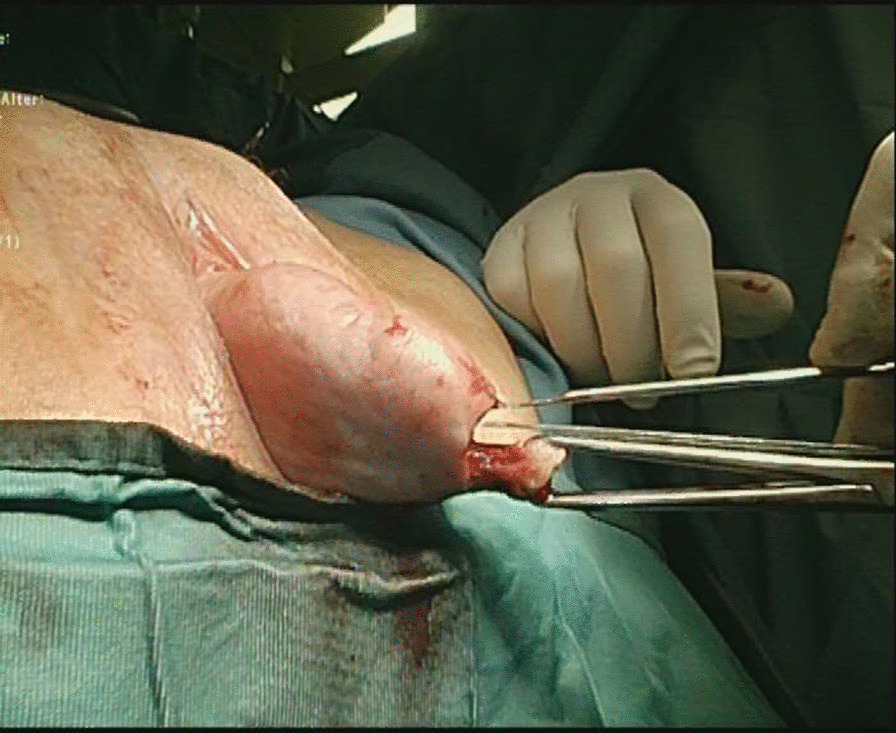
Stage IV uterovaginal prolapse

**Fig. 5:**
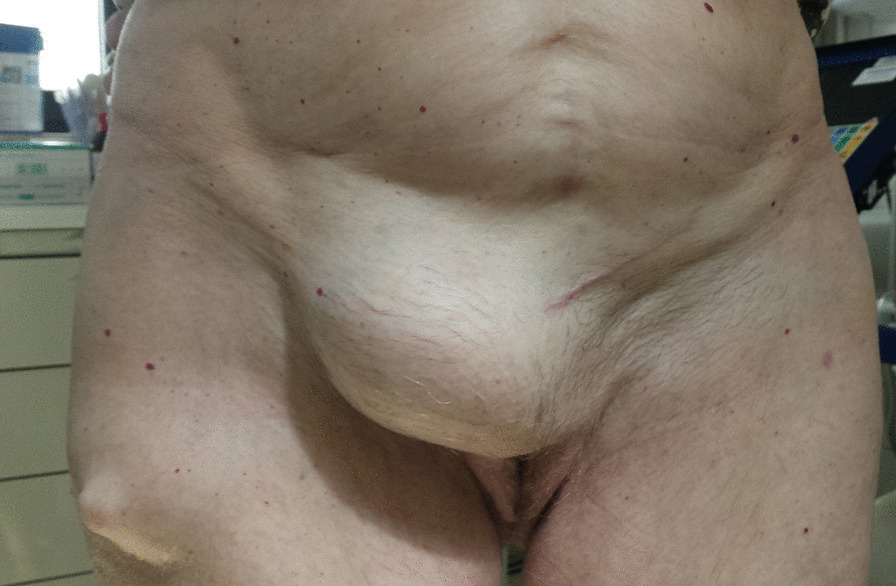
Inguinal bladder hernia at follow up 6 months after reconstructive surgery for POP

An 80-year-old woman presented with stage IV uterovaginal prolapse in January 2015 and underwent vaginal native-tissue reconstructive surgery (Fig. 4). Six months later, an MIII inguinal hernia (Aachen classification [15]) was found to have developed. The hernia sac contained large parts of the urinary bladder, which had herniated through the inguinal hernia orifice (Fig. 5).

### Case 3

**Fig. 6:**
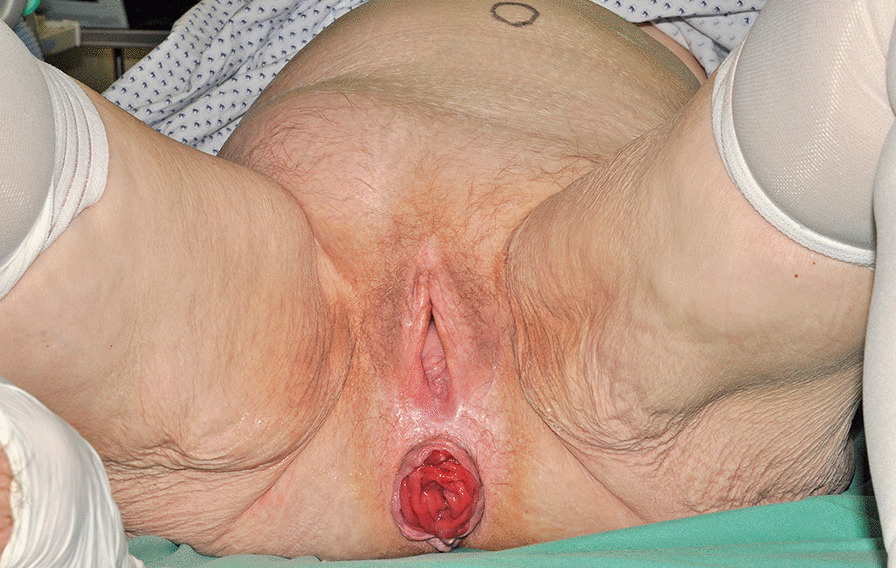
Simultaneous finding of POP, rectal prolapse and inguinal hernia

An 81-year-old women presented in February 2015 with the simultaneous finding of POP, rectal prolapse and inguinal hernia (Fig. 6). A follow-up examination conducted 6 months later revealed no change. On ultrasound, the hernia sac was found to contain the urinary bladder having herniated through the inguinal hernia orifice, and the diagnosis of bladder hernia was established.

## Discussion and conclusion

In all three cases reported here, the patients were of advanced age with long-standing menopausal status. In the context of demographic development in industrialised countries, many elderly adults are in good general condition and have high expectations for their quality of life. Thus, we find it important to discuss surgery in the context of advanced age and ageing.

In two of the cases presented here, MIII inguinal hernias developed or enlarged significantly following reconstructive surgery for POP. One patient presented with the triple finding of POP, rectal prolapse and inguinal hernia. In all cases, ultrasound examination revealed that the hernia sacs contained the urinary bladder, which had herniated through the inguinal hernia orifice.

Inguinal bladder hernia is rare [2]. Levine [16] first described it in 1951, using the term “scrotal cystocele”. It usually occurs in men with other risk factors, such as obesity and/or age > 50 years [3]. Less than 7% of such hernias are diagnosed prior to surgery due to their asymptomatic presentation or nonspecific symptoms, such as haematuria, dysuria, urinary urgency and inguinal swelling [17]. The literature contains several case reports and case series of inguinal bladder hernia in men [2, 3]. Alsayegh et al. [18] indicated that about 150 such cases had been reported through 2004. Branchu et al. [19] included 64 cases of inguinal bladder hernia in men in their systematic review covering the period 2005–2017. One case of indirect inguinal bladder hernia in a female patient, detected post-mortem, was reported by Tubbs et al. [20]. A search of the PubMed database using the terms “inguinal hernia” AND “bladder” AND “female” or “inguinal bladder hernia” AND “female” revealed one additional case of direct inguinal bladder hernia in a 72-year-old woman, reported in 2018 by Caliscan et al. [4]. In two of our presented cases, herniation had developed or enlarged following reconstructive surgery for POP due to multiple conditions and into an anatomical musculoaponeurotic weakness—a “locus minoris resistentiae”.

Although reports on this type of bladder herniation are rare [4], the simultaneous finding of inguinal hernia and POP may be more common, as both conditions are considered to be associated with connective tissue weakness [12, 13]. Few studies of POP risk constellations have examined this simultaneous presentation [5, 9–11]. Risk factors for POP include menopausal status, advanced age, high body mass index, difficult obstetric history, spontaneous vaginal deliveries, heavy labour, family history of prolapse and signs of connective tissue disorders (e.g. varicose veins, haemorrhoids and hernia) [8, 9]. Previous hernia surgery was examined only in one of the studies included in a review of risk factors for POP and its recurrence; the authors thus stated that this factor could not be associated with primary POP without additional confirmatory evidence [9]. However, in a cross-sectional study including 1380 women in The Netherlands, previous surgery for POP and/or incontinence was associated with previous hernia surgery [10]. A case–control study showed that inguinal hernia was significantly more prevalent among 60 patients with advanced (stage III–IV) POP than among 60 patients with no or mild (stage 0–I) POP [11]. However, comprehensive data on the co-existence of hernia and female genital prolapse remain scarce.

A historical review of the literature of the past 40 years confirmed that similar pathophysiological mechanisms underlie POP, inguinal hernia and abdominal wall defects referred to as “herniosis”, which could be responsible for the co-occurrence of the conditions [11, 21]. An increasing number of studies has revealed collagen alterations associated with POP and hernia [12, 13]. Changes in the quantity and ratio of collagen subtypes I and III, biomechanical properties and the structure of collagen fibrils, and abnormalities in collagen catabolism, caused for example by imbalances of matrix metalloproteinases and tissue inhibitors of metalloproteinases, have been described [12, 13]. Furthermore, collagen gene alterations have been associated with the development of inguinal hernia and POP [13, 22, 23]. Tissue ageing is reported to be an important endogenic trigger of both conditions [9, 24].

The simultaneous occurrence of hernia and female genital prolapse may also be conceptualised according to the “locus minoris resistentiae” model. This model is used in diverse fields of medicine, such as dermatology and internal medicine [14, 25]. Siegfried´s shoulder and Achilles’ heel are the original “locus minoris resistentiae” examples from Greek mythology [14]. In medicine, the term refers to any part of the body that is more vulnerable than other regions to disease due to altered firmness [25]. In anatomy, it describes regions in the musculoaponeurotic system with lesser resistance, leading to herniation, such as the female pelvic floor and the inguinal canal [26]. In our cases, the bladder had found its way to a second “locus minoris resistentiae” after the first such location had been closed surgically.

In summary, although reports on inguinal bladder hernia in women are rare, the simultaneous occurrence of hernia orifices and POP might be more common, as evidence suggests that hernia and POP co-exist frequently [4, 9–11] and that similar collagen alterations underlie both conditions [12, 13]. With this report, we present our observations on a small sample of cases of this rare condition. A larger prospective study on the topic employing the “locus minoris resistentiae” model for the examination of detailed preoperative and follow-up information on patients’ histories, risk factors for POP or hernia and individual complaints, is warranted. With additional evidence, the simultaneous finding of hernia and POP could be discussed further as a valid clinical marker for the future definition of connective tissue weakness for clinical assessment*.*

The simultaneous occurrence of inguinal hernia and female POP can lead to bladder hernia after prolapse surgery, in the sense of a “locus minoris resistentiae”. Clinical examination for simultaneous signs of connective tissue weakness and counselling prior to pelvic reconstructive surgery could help to increase patients’ compliance with further surgical treatment for hernia.

## Data Availability

The data supporting the conclusions of this article is available from corresponding author.

## Abbreviation

POP: Pelvic organ prolapse
